# Co-production of acetoin and succinic acid by metabolically engineered *Enterobacter cloacae*

**DOI:** 10.1186/s13068-021-01878-1

**Published:** 2021-01-19

**Authors:** Hsiang-Yen Su, Hua-Ying Li, Cai-Yun Xie, Qiang Fei, Ke-Ke Cheng

**Affiliations:** 1 Engineering Research Center of Health Food Design & Nutrition Regulation, School of Chemical Engineering and Energy Technology, Dongguan University of Technology, Dongguan, 523808 China; 2 School of Chemical Engineering and Technology, Xi’an Jiaotong University, Xi’an, 710049 China; 3 China-Latin America Joint Laboratory for Clean Energy and Climate Change, School of Chemical Engineering and Energy Technology, Dongguan University of Technology, Dongguan, 523808 China

**Keywords:** *Enterobacter cloacae*, Metabolic engineering, Co-production, Acetoin, Succinic acid

## Abstract

**Background:**

Renewable chemicals have attracted attention due to increasing interest in environmental concerns and resource utilization. Biobased production of industrial compounds from nonfood biomass has become increasingly important as a sustainable replacement for traditional petroleum-based production processes depending on fossil resources. Therefore, we engineered an *Enterobacter cloacae budC* and *ldhA* double-deletion strain (namely, EC∆budC∆ldhA) to redirect carbon fluxes and optimized the culture conditions to co-produce succinic acid and acetoin.

**Results:**

In this work, *E. cloacae* was metabolically engineered to enhance its combined succinic acid and acetoin production during fermentation. Strain EC∆budC∆ldhA was constructed by deleting 2,3-butanediol dehydrogenase (*budC*), which is involved in 2,3-butanediol production, and lactate dehydrogenase (*ldhA*), which is involved in lactic acid production, from the *E. cloacae* genome. After redirecting and fine-tuning the *E. cloacae* metabolic flux, succinic acid and acetoin production was enhanced, and the combined production titers of acetoin and succinic acid from glucose were 17.75 and 2.75 g L^−1^, respectively. Moreover, to further improve acetoin and succinic acid production, glucose and NaHCO_3_ modes and times of feeding were optimized during fermentation of the EC∆budC∆ldhA strain. The maximum titers of acetoin and succinic acid were 39.5 and 20.3 g L^−1^ at 72 h, respectively.

**Conclusions:**

The engineered strain EC∆budC∆ldhA is useful for the co-production of acetoin and succinic acid and for reducing microbial fermentation costs by combining processes into a single step.

## Background

Renewable chemicals have attracted attention due to increasing interest in environmental concerns and resource utilization. Biobased production of industrial compounds from nonfood biomass has become increasingly important as a sustainable replacement for traditional petroleum-based production processes depending on fossil resources. Both acetoin and succinic acid are C4 chemicals that listed biobased high-value-added chemicals by the United States Department of Energy [1]. Currently, acetoin and succinic acid are building block chemicals used extensively in the food and pharmaceutical industries.

Acetoin is a volatile compound that occurs naturally in certain fruits and dairy products. Commercial acetoin can be used as a plant growth promoter, biological pest control measure, and additive to improve food flavor [2, 3]. At present, acetoin is produced mainly by chemical synthetic routes. Compared with chemical synthesis methods, microbial fermentation methods have the advantages of easy access to feedstock, environmental friendliness and mild process conditions [4, 5]. Therefore, microbial fermentation is considered to be an environmentally friendly method for the production of acetoin, which has made great progress in recent years. Many microorganisms synthesize acetoin during the mixed acid fermentation process, such as *Enterobacter*, *Klebsiella*, *Lactococcus*, *Bacillus*, *Serratia* and *Saccharomyces* species [6]. Acetoin is an intermediate product of the 2,3-butanediol biosynthesis pathway [7]. It is produced from pyruvate through α-acetolactate by two enzymes, including α-acetolactate synthase (*budB*) and α-acetolactate decarboxylase (*budA*), and finally converted to 2,3-butanediol by 2,3-butanediol dehydrogenase (*budC*) with the consumption of NADH [8]. Several studies have reported that the deletion of 2,3-butanediol dehydrogenase (*budC*) improves the production of acetoin in different species of microorganisms [2, 3, 9, 10].

In a traditional acetoin fermentation process, succinic acid is an undesirable by-product. However, succinic acid, a C4 dicarboxylic acid, has been used as a precursor for various chemicals, ion chelators, and additives in the food and pharmaceutical industries [11]. In addition, succinic acid can be converted into other chemicals, such as γ-butyrolactone, 1,4-butanediol, and tetrahydrofuran, and act as the precursor of polybutylene succinate synthesis. In *E. coli,* the reductive branch of the tricarboxylic acid (TCA) pathway function is the key pathway for the synthesis of succinic acid. The carboxylation of phosphoenolpyruvate (PEP) to oxaloacetate (OAA) catalyzed by PEP carboxylase (PEPC) is considered the most important reaction. In this step, 1 mol CO_2_ is assimilated to form OAA [2]. Therefore, CO_2_ is an essential substrate for succinic acid biosynthesis, and it has been demonstrated that the production of succinic acid can be increased by sodium bicarbonate (NaHCO_3_) addition to the culture medium [12, 13]. Additionally, when grown under anaerobic conditions, *E. coli* metabolizes phosphoenolpyruvate (PEP) and pyruvate via the glycolytic pathway to form ethanol, lactic acid, and formic acid [14]. Therefore, changing the carbon flux towards the synthesis of succinic acid by metabolic engineering is very important [15]. Currently, numerous industrially used microorganisms have been metabolically engineered for succinic acid production by fermentation [16–19].

**Fig. 1:**
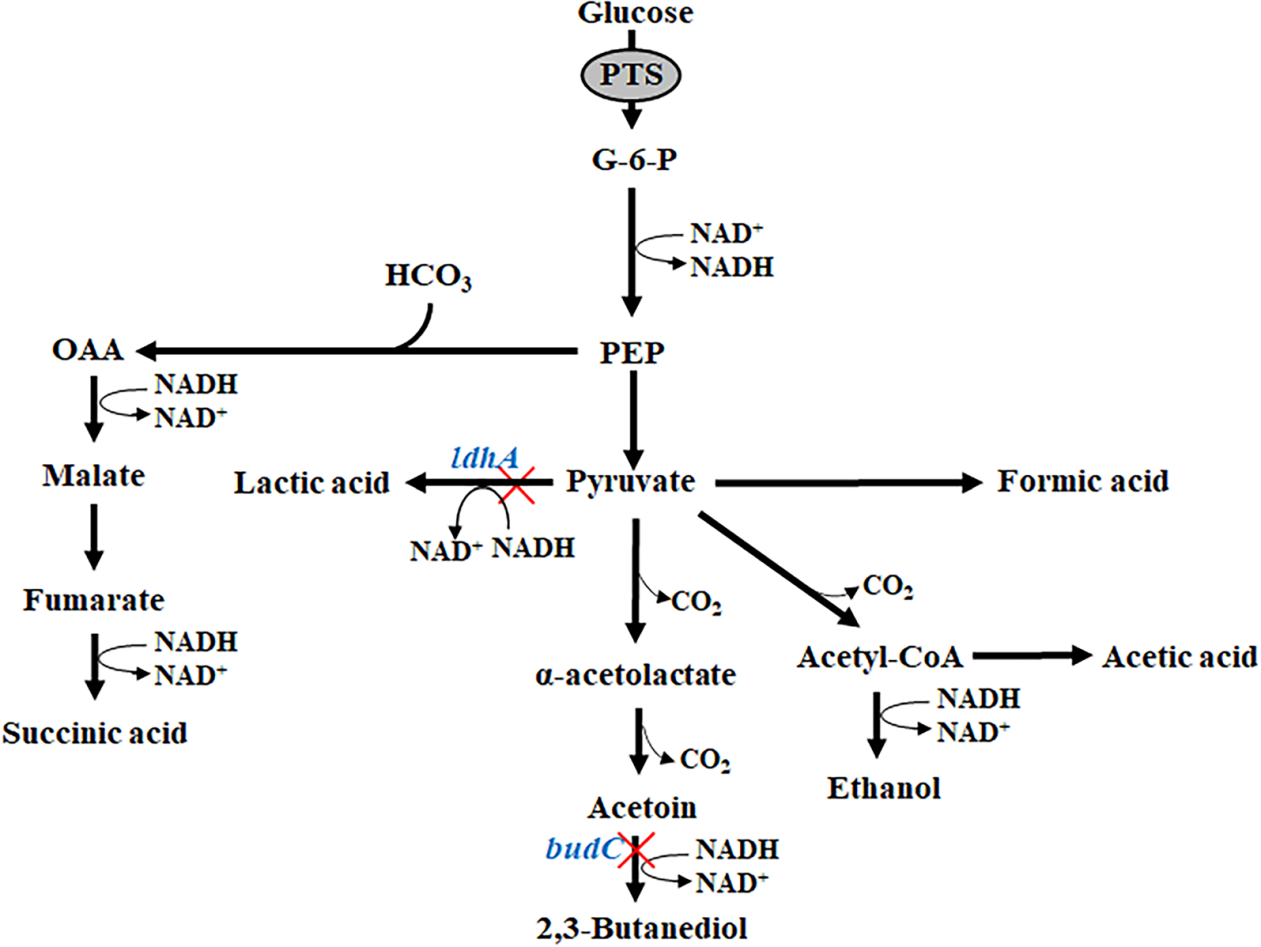
Schematic representation of improved production of acetoin and succinic acid by deletion of *budC* and *ldhA* in *E. cloacae*

An earlier study revealed that *E. cloacae* can produce 40.67 g L^−1^ 2,3-butanediol and 21.79 g L^−1^ succinic acid from xylose [20]. In this study, to redirect the carbon flux, a double-deletion mutant (strain EC∆budC∆ldhA) of *E. cloacae* was developed by deleting 2,3-butanediol dehydrogenase (*budC*) to produce acetoin and by deleting lactate dehydrogenase (*ldhA*) to improve succinic acid production (Fig. 1). Moreover, the feeding mode and time of glucose and NaHCO_3_ during the fermentation of the EC∆budC∆ldhA strain were optimized, which further enhanced the production of acetoin and succinic acid. The present findings demonstrated a potential practical strategy for the simultaneous production of two commercial products in a single fermentation step by redirecting the carbon flux and optimizing the culture conditions.

## Results

### Construction of the metabolically engineered strains

*Enterobacter cloacae* has an extraordinary ability to utilize biomass for 2,3-butanediol production, during which intermediary acetoin is formed [10]. Acetoin reductases (also known as 2,3-butanediol dehydrogenase) catalyze the transformation reaction from acetoin to 2,3-butanediol. Knockout of the *budC* gene would generate the EC∆budC strain, which mainly produces acetoin. A previous study showed that reducing the carbon flux to lactate, ethanol, and acetate by-products can be performed by deleting the *ldhA*, *adhE*, and *pta* genes in *K. pneumoniae* [7]. In this study, to further reduce lactic acid production in the fermentation process, the *ldhA* gene was disrupted from the wild type and EC∆budC strains to generate strains EC∆ldhA and EC∆budC∆ldhA, respectively. The *budC* and *ldhA* gene knockout of these strains was verified by screening with colony PCR (data not shown).

**Fig. 2:**
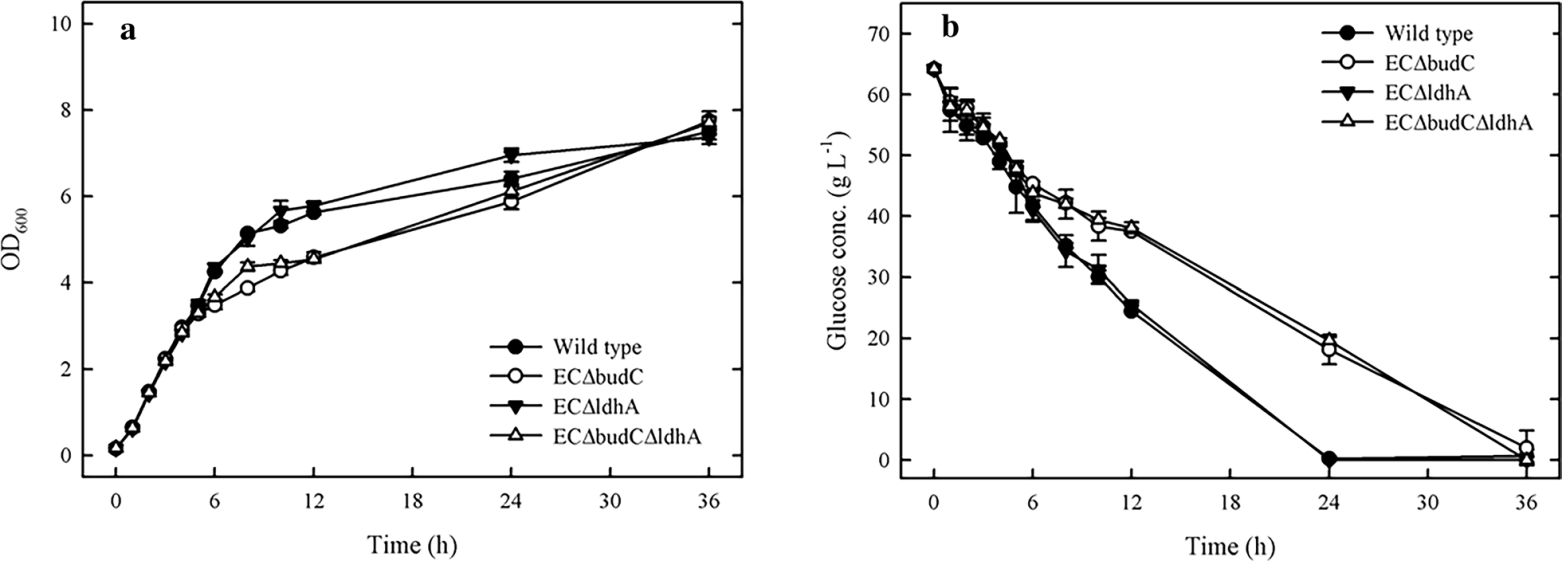
Effects of knockout *budC* and *ldhA* on cell growth and consumed glucose of *E. cloacae*. The experiments were conducted in 50 mL of fermentation medium containing 60 g L^−1^ glucose and 5.0 g L^−1^ NaHCO_3_ in a 250 mL flask at 35 °C with shaking (150 rpm)

To determine the effects of deleting *budC* and *ldhA* on cell growth, wild type and three deletion mutants, EC∆budC, EC∆ldhA, and EC∆budC∆ldhA, were grown under 60 g L^−1^ glucose and 5 g L^−1^ NaHCO_3_ at 35 °C at 150 rpm, followed by comparison of the growth curves. The initial inoculum of these cultures was the same (OD_600_ = 0.15). The growth results are shown in Fig. 2. The EC∆budC and EC∆budC∆ldhA strains grew slower than the wild type in the first 12 h. The OD_600_ values were 5.62, 4.58, and 4.54 for the wild type, EC∆budC, and EC∆budC∆ldhA strains, respectively, after 24 h (Fig. 2). The growth rates of the EC∆budC and EC∆budC∆ldhA strains were reduced by 22.7% and 23.7%, respectively, in comparison with the wild type. In contrast, compared with the wild type, the EC∆ldhA strain exhibited increased cell growth. The OD_600_ value was 5.77 for the EC∆ldhA strain (Fig. 2a). The glucose concentration in the medium of wild type and EC∆ldhA strains was depleted after 24 h of fermentation. The glucose concentrations of EC∆budC and EC∆budC∆ldhA were depleted at 36 h (Fig. 2b). Jang et al. [9] reported that deletion of *budC* resulted in reduced cell growth and glucose consumption rate in *Enterobacter aerogenes*. These results indicated that deletion of the *budC* gene in *E. cloacae* cells might reduce the growth rate and glucose consumption.

### Effects of metabolic engineering on enhanced co-production of acetoin and succinic acid

The wild type and EC∆budC strains were grown at 35 °C in 250 mL shake flasks containing 50 mL of fermentation medium supplemented with 90.0 g L^−1^ glucose and 5.0 g L^−1^ NaHCO_3_. The fermentation was finished when glucose was consumed nearly completely. As shown in Table 1, EC∆budC produced various organic acids and ethanol, with acetoin being a major product that accumulated to 18.6 g L^−1^, resulting in a 0.419 yield (mol mol^−1^ glucose). The concentrations of succinic acid, 2,3-butanediol, lactic acid, acetic acid and ethanol were 1.05, 7.7, 2.6, 2.75, and 4.75 g L^−1^, respectively. The succinic acid content of the EC∆budC strain was decreased by 2.24-fold in comparison with that of the wild type strain. Succinic acid was measured as 2.35 and 1.05 for the wild type and EC∆budC strains, respectively. Lactic acid formation was not detected in the wild type strain. However, the lactic acid content of EC∆budC (2.6 g L^−1^) was increased compared with that of the control and wild type (not detected).

**Table 1:** Fermentation profiles of the gene knockout strains of *E. cloacae*

Strain	WT	∆budC	∆ldhA	∆budC∆ldhA
Final pH	5.92 ± 0.007	6.08 ± 0.071	6.09 ± 0.0035	5.9 ± 0.028
Consumed glucose (g L^−1^)	92.2 ± 0	90.8 ± 0	92.7 ± 0	91.4 ± 4.34
Final OD_600_	8.92 ± 0.057	7.83 ± 0.028	9.345 ± 0.274	8.245 ± 0.12
Final succinic acid (g L^−1^)	2.35 ± 0.071	1.05 ± 0.071	2.8 ± 0.141	2.75 ± 0.071
Final acetoin (g L^−1^)	1.25 ± 0.071	18.6 ± 0.99	1.35 ± 0.212	17.75 ± 0.354
Final 2,3-BDO (g L^−1^)	28.75 ± 0.212	7.7 ± 0.849	29.05 ± 0.212	8.15 ± 0.071
Final lactic acid (g L^−1^)	ND	2.6 ± 0.283	ND	ND
Final acetic acid (g L^−1^)	1.3 ± 0.283	2.75 ± 0.071	1.3 ± 0.00	1.4 ± 0.141
Final ethanol (g L^−1^)	5.0 ± 0.141	4.75 ± 0.778	5.05 ± 0.354	5.05 ± 0.212
Lactic acid (mol mol^−1^)	ND	0.057 ± 0.006	ND	ND
Succinic acid yield (mol mol^−1^)	0.039 ± 0.001	0.018 ± 0.001	0.046 ± 0.002	0.046 ± 0.001
Acetoin yield (mol mol^−1^)	0.028 ± 0.002	0.419 ± 0.022	0.03 ± 0.005	0.397 ± 0.008
2,3-BDO yield (mol mol^−1^)	0.623 ± 0.005	0.170 ± 0.019	0.626 ± 0.005	0.178 ± 0.002

Each date indicates the mean ± SD from two experiments

*ND* not detected

d-Lactate dehydrogenase (encoded by *ldhA*) catalyzes the conversion of pyruvate to d-lactic acid by coupling with the oxidation of NADH in *E. cloacae* [21]. In this study, to reduce lactic acid production in the fermentation process, the *ldhA* gene was inactivated in the wild type. The results indicated that the fermentation products of the EC∆ldhA strain were similar to those of the wild type (Table 1). The difference is that the succinic acid content of the EC∆ldhA strain was increased by 19% in comparison with that of the wild type.

To achieve a higher yield of acetoin and succinic acid co-production, the EC∆budC∆ldhA strain was constructed by knocking out *ldhA* genes in strain EC∆budC. EC∆budC∆ldhA produced various organic acids and ethanol, with acetoin being a major product that accumulated to 17.75 g L^−1^, resulting in a yield of 0.397 (mol mol^−1^ glucose). The concentrations of succinic acid, 2,3-butanediol, acetic acid and ethanol were 2.75, 8.15, 1.4, and 5.05 g L^−1^, respectively. Lactic acid was not observed in the EC∆budC∆ldhA strain. The succinic acid content of the EC∆budC∆ldhA strain was increased by 2.24-fold in comparison with that of the EC∆budC strain. The final concentrations of succinic acid were measured as 1.05 and 2.75 g L^−1^ for the EC∆budC and EC∆budC∆ldhA strains, respectively. The succinic acid yield was measured as 0.046 and 0.018 (mol mol^−1^ glucose) for the EC∆budC∆ldhA and EC∆budC strains, respectively.

The results indicated that elimination of 2,3-butanediol and lactic acid formation in the EC∆budC∆ldhA strain led to enhanced acetoin and succinic acid co-production, and the maximum acetoin and succinic acid yields were obtained as 0.397 and 0.046 mol mol^−1^ glucose, respectively.

### Effect of NaHCO_3_ concentration on metabolite production by the EC∆budC∆ldhA strain

Previous studies have shown that CO_2_ is a key parameter in batch succinic acid fermentation. The amount of dissolved CO_2_ can be increased effectively by the addition of NaHCO_3_ to the medium [20]. To compare the effects of NaHCO_3_ levels, different concentrations of NaHCO_3_ (0, 2.5, 5, 7.5, and 10 g L^−1^) were added to the fermentation medium. As shown in Table 2, when grown in fermentation medium without NaHCO_3_, the final production of acetoin after 24 h was 16.45 g L^−1^. The concentrations of succinic acid, 2,3-butanediol and ethanol were 1.15, 3, and 6.25 g L^−1^, respectively. However, a higher concentration of NaHCO_3_ led to a negative effect on acetoin production. The concentration of acetoin was decreased by 7, 19, 10.6, and 24.6% for 2.5, 5, 7.5, and 10 g L^−1^ NaHCO_3_ addition, respectively. When grown in fermentation medium supplemented with different NaHCO_3_ levels (0, 2.5, 5, 7.5, and 10 g L^−1^), the concentrations of succinic acid were slightly enhanced from 1.15 to 1.55 g L^−1^ within 24 h. The maximum acetoin and succinic acid yields (0.469 and 0.034 mol mol^−1^ glucose) were obtained when 2.5 g L^−1^ NaHCO_3_ was added. Furthermore, the cell growth and acetic acid titer were also improved, while the amount of 2,3-butanediol slightly decreased. Therefore, the optimum NaHCO_3_ concentration for the combined production of acetoin and succinic acid was 2.5 g L^−1^.

**Table 2:** The fermentation performance of EC∆budC∆ldhA strain under different NaHCO_3_ concentration

NaHCO_3_ conc. (g L^−1^)	0	2.5	5	7.5	10
Final pH	5.43 ± 0.58	5.92 ± 0.021	5.94 ± 0.191	6.2 ± 0.127	6.56 ± 0.021
Consumed glucose (g L^−1^)	67.45 ± 1.626	66.7 ± 4.808	61 ± 3.111	64.65 ± 4.879	67 ± 0.4.667
Final OD_600_	4.21 ± 0.078	5.155 ± 0.007	5.305 ± 0.064	5.84 ± 0.113	5.79 ± 0.226
Final succinic acid (g L^−1^)	1.15 ± 0.212	1.5 ± 0.283	1.4 ± 0.141	1.45 ± 0.212	1.55 ± 0.212
Final acetoin (g L^−1^)	16.45 ± 0.212	15.3 ± 1.273	13.3 ± 0.707	14.7 ± 1.414	12.4 ± 1.556
Final 2,3-BDO (g L^−1^)	3 ± 0.283	2.65 ± 0.212	2 ± 0.141	2.7 ± 0.283	1.95 ± 0.495
Final lactic acid (g L^−1^)	ND	ND	ND	ND	ND
Final acetic acid (g L^−1^)	ND	ND	0.9 ± 0	1.2 ± 0	1.5 ± 0.141
Final ethanol (g L^−1^)	6.25 ± 0.354	6.8 ± 0.283	6.15 ± 0.212	6.05 ± 0.212	6.1 ± 0.566
Succinic acid yield (mol mol^−1^)	0.026 ± 0.004	0.034 ± 0.004	0.032 ± 0.02	0.034 ± 0.02	0.036 ± 0.01
Acetoin yield (mol mol^−1^)	0.499 ± 0.018	0.469 ± 0.005	0.446 ± 0.001	0.465 ± 0.01	0.381 ± 0.028
2,3-BDO yield (mol mol^−1^)	0.089 ± 0.011	0.079 ± 0.001	0.066 ± 0.001	0.083 ± 0.002	0.059 ± 0.019

Each date indicates the mean ± SD from two experiments

*ND* not detected

### Fed-batch fermentation for co-production of acetoin and succinic acid

To increase the production of acetoin and succinic acid, fed-batch fermentation was performed using strain EC∆budC∆ldhA with an initial glucose concentration of 57.8 g L^−1^. NaHCO_3_ (1 g L^−1^) was added at fermentation times of 6 and 12 h. Next, glucose (20 g L^−1^) and NaHCO_3_ (2 g L^−1^) were added simultaneously at 24, 36, 48, 60, 72, and 84 h of fermentation.

**Fig. 3:**
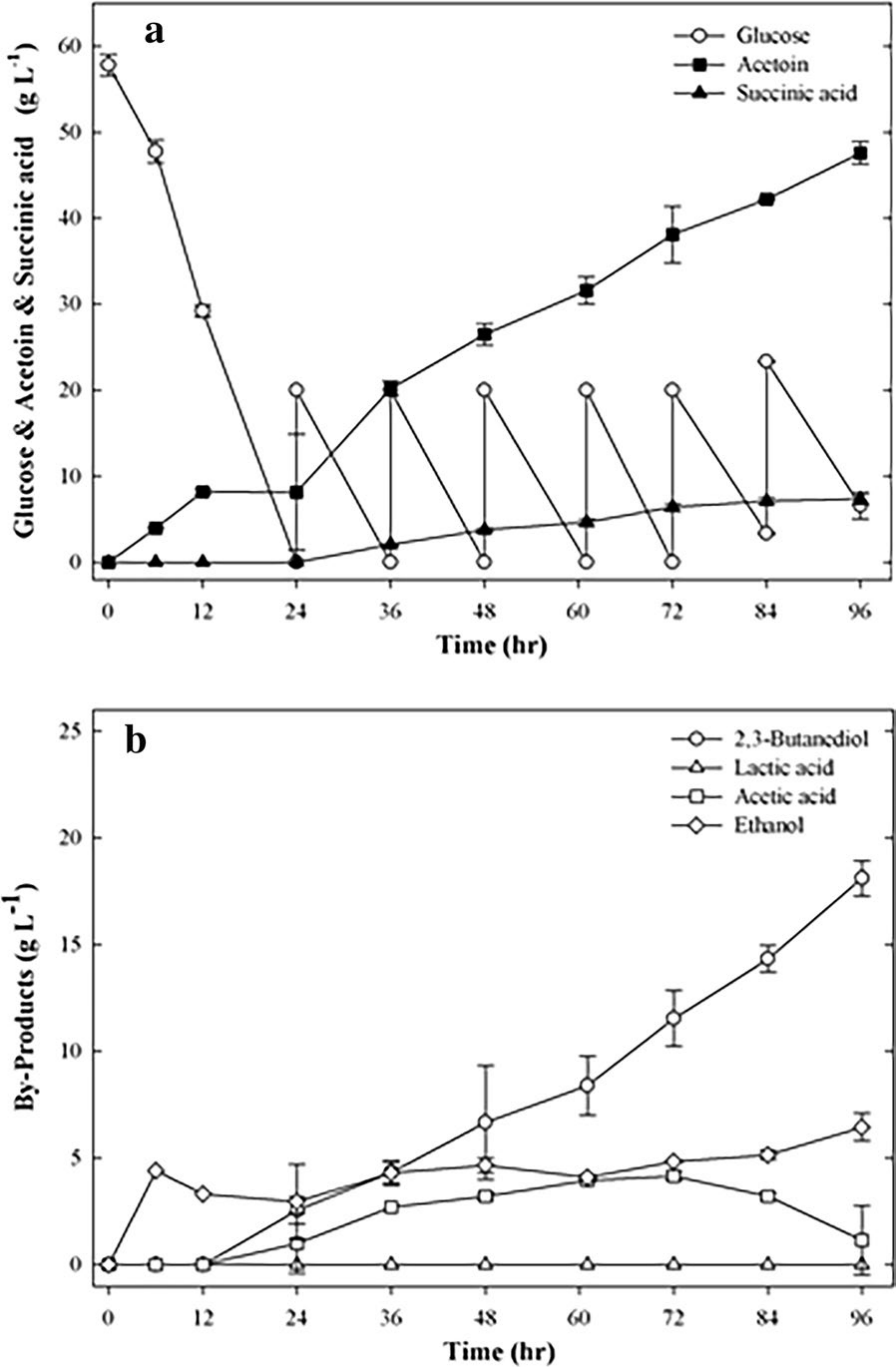
Time course of fed-batch fermentation of EC∆budC∆ldhA. **a** Glucose, acetoin, succinic acid, **b** by-product. The experiments were conducted in 50 mL of fermentation medium containing 60 g L^−1^ glucose in a 250 mL flask. NaHCO_3_ (1 g L^−1^) was added at 6 and 12 h of fermentation. After that, NaHCO_3_ (2 g L^−1^) was added at 24, 36, 48, 61, 72, and 84 h of fermentation. Glucose (20 g L^−1^) was added at 24, 36, 48, 61, 72, and 84 h of fermentation. Samples were withdrawn every 12 h for detection of cell density and concentration of substrates and products

Figure 3 shows 47.6 g L^−1^ acetoin and 7.35 g L^−1^ succinic acid from 171.25 g L^−1^ glucose obtained in 96 h by the EC∆budC∆ldhA strain. The acetoin and succinic acid yields were 0.568 and 0.065 mol mol^−1^ glucose, respectively. In fed-batch fermentation, the maximum acetoin and succinic acid yields were obtained as 0.565 and 0.071 mol mol^−1^ glucose, respectively, after 72 h. Compared with batch fermentation (Table 2), the maximum acetoin and succinic acid yields of fed-batch fermentation were increased by 1.2- and 2-fold, respectively. The results indicated that the acetoin and succinic acid co-production of the EC∆budC∆ldhA strain was improved by fed-batch fermentation.

### Optimization of fed-batch fermentation for co-production of acetoin and succinic acid

**Fig. 4:**
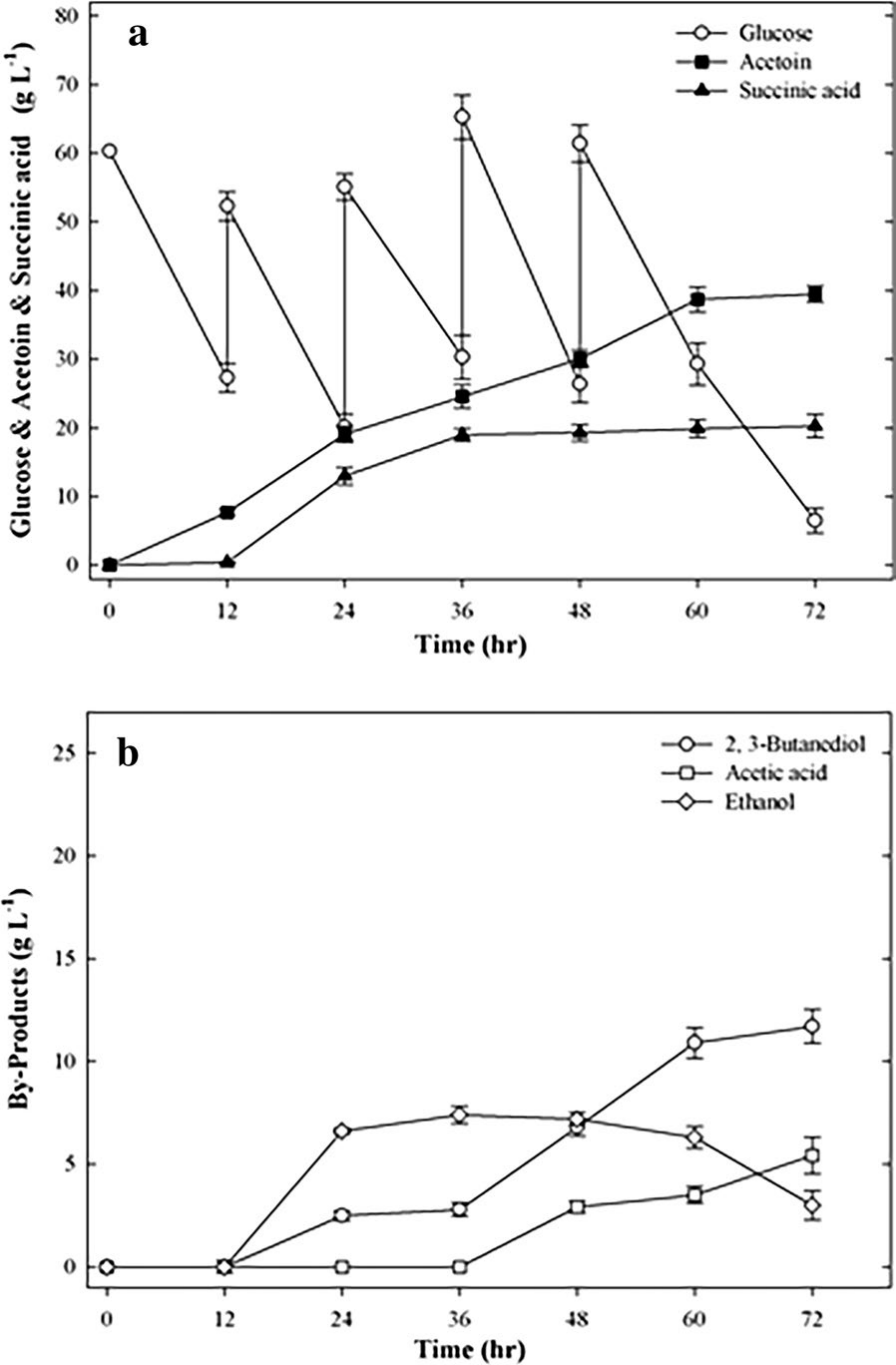
Time course of acetoin and succinic acid co-production by fed-batch fermentation using strain EC∆budC∆ldhA under the optimized conditions. **a** Glucose, acetoin, succinic acid, **b** by-product. The experiments were conducted in 50 mL of fermentation medium containing 60 g L^−1^ glucose in a 250 mL flask. NaHCO_3_ (2.5, 1, 1, 5, 5, 5 and 2 g L^−1^) was added at 0, 6, 12, 24, 36, 48, and 60 h of fermentation, respectively. Glucose (60, 25, 35, 35, and 35 g L^−1^) was added at 0, 12, 24, 36, and 48 h of fermentation, respectively. Samples were withdrawn every 12 h for detection of cell density and concentration of substrates and products

A previous study showed that after glucose was depleted, the accumulated products were reused by *Klebsiella pneumoniae* as a carbon source [2]. Our previous work also found that with a low glucose concentration during cultivation, succinic acid did not accumulate (data not shown). To prevent the exhaustion of glucose, the glucose concentration was increased during fed-batch fermentation to achieve higher acetoin and succinic acid co-production. In this study, the optimal conditions for acetoin and succinic acid fermentation were determined by the glucose and NaHCO_3_ times and modes of feeding. The initial glucose and NaHCO_3_ concentrations were 60 g L^−1^ and 2.5 g L^−1^, respectively. When the glucose concentration was reduced to approximately 30 g L^−1^, glucose was added to the fermentation medium. Glucose (25, 35, 35 and 35 g L^−1^) was added at 12, 24, 36 and 48 h of fermentation, respectively. NaHCO_3_ (1, 1, 5, 5, 5, and 2 g L^−1^) was added at 6, 12, 24, 36, 48, and 60 h of fermentation, respectively. The time-course results of the production of succinic acid are shown in Fig. 4. After growth for 72 h, the maximum production of acetoin and succinic acid was measured as 39.5 g L^−1^ and 20.3 g L^−1^, respectively, in optimized fed-batch fermentation. The acetoin and succinic acid yields were 0.439 and 0.168 mol mol^−1^ glucose, respectively. The maximum acetoin and succinic acid yields were obtained as 0.559 and 0.322 mol mol^−1^ glucose, after 36 h. Compared with fed-batch fermentation (Fig. 3), the succinic acid titer of optimized fed-batch fermentation was increased by 2.8-fold. However, when only the *budC* gene was deleted, we found that a large amount of lactic acid (15 g L^−1^) was produced under the same optimized conditions, and caused a decrease in the production of succinic acid (Additional file 1: Fig. S1). This is similar to the results in Table 1. Thus, deletion of the *ldhA* gene of *E. cloacae* is required. The results indicated that the succinic acid production of the EC∆budC∆ldhA strain was significantly increased by optimizing the glucose and NaHCO_3_ feeding mode and time during fermentation, further enhancing the co-production concentrations of acetoin and succinic acid.

### Fed-batch fermentation for co-production of acetoin and succinic acid in a bioreactor

Previous studies have shown that cultivating the EC∆budC∆ldhA strain in a 250 mL flask through an optimized fed-batch culture method can increase the production of acetoin and succinic acid. Therefore, the same optimized fed-batch fermentation conditions were implemented in a 3-L bioreactor.

**Fig. 5:**
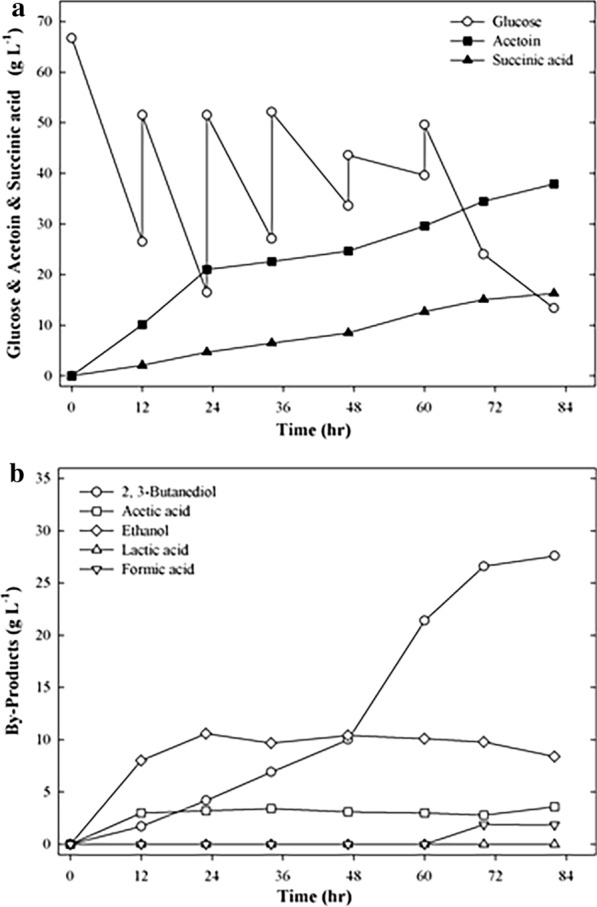
Time course of acetoin and succinic acid co-production by fed-batch fermentation using strain EC∆budC∆ldhA in bioreactor. **a** Glucose, acetoin, succinic acid, **b** by-product. The experiments were conducted in 1.5-L of fermentation medium containing 60 g L^−1^ glucose under 0.5 vvm air flow in 3-L bioreactor. NaHCO_3_ (2.5, 1, 1, 5, 5, 5, and 2 g L^−1^) was added at 0, 6, 12, 24, 36, 48, and 60 h of fermentation, respectively. Glucose (60, 25, 35, 25, 10, and 10 g L^−1^) was added at 0, 12, 24, 36, 48, and 60 h of fermentation, respectively. Samples were withdrawn every 12 h for detection of cell density and concentration of substrates and products

As shown in Fig. 5, the initial glucose and NaHCO_3_ concentrations were 60 g L^−1^ and 2.5 g L^−1^, respectively. Glucose (25, 35, 25, 10, and 10 g L^−1^) was added at 12, 24, 36, 48, and 60 h of fermentation, respectively. NaHCO_3_ (1, 1, 5, 5, 5, and 2 g L^−1^) was added at 6, 12, 24, 36, 48, and 60 h of fermentation, respectively. The time-course results are shown in Fig. 5. After growth for 82 h, the maximum production of acetoin and succinic acid was measured as 38 g L^−1^ and 16.3 g L^−1^, respectively. The acetoin and succinic acid yields were 0.490 and 0.157 mol mol^−1^ glucose, respectively.

## Discussion

Several studies have also shown that the inactivation of *budC* significantly improves the production of acetoin. Indeed, previous reports have shown that the deletion of the *budC* gene could decrease 2,3-butanediol. Three butanediol stereoisomers, namely, (2R,3R)-2,3-butanediol, (2S,3S)-2,3-butanediol, and meso-2,3-butanediol, are found in many bacterial species, such as *Enterobacter cloacae* [10, 22], *Klebsiella pneumoniae* [23], and *Bacillus licheniformis* [24], and meso-2,3-butanediol and (2S,3S)-2,3-butanediol are the major forms that accumulate in *E. cloacae* [25]. However, when the *budC* gene was deleted, a small amount of 2,3-butanediol could still be detected [3, 22, 23, 26, 27]. In this study, the *budC* gene was knocked out, and we observed that the production of meso-2,3-butanediol and (2S,3S)-2,3-butanediol decreased (data not shown).

A previous study characterized a *budC* and glycerol dehydrogenase (encoded by *gldA* and *dhaD*)-deficient *Klebsiella pneumoniae* strain*,* which removes 2,3-butanediol under conditions wherein glycerol is used as a carbon source. These findings suggested that *dhaD* and *gldA* may be involved in 2,3-butanediol formation [22]. Another study reported diacetyl production by inactivating *budA*, *budC*, and diacetyl reductases (also known as glycerol dehydrogenase, encoded by *gdh*) in *E. cloacae* SDM. When the *gdh* and *budC* genes were both inactivated in the strain *E. cloacae* SDM (∆budA), (2R,3R) 2,3-butanediol could be slightly detected [10]; these results show that there is a third enzyme responsible for 2,3-butanediol production in the *E. cloacae* strain. In the present work, disruption of the *budC* gene remarkably decreased the production of 2,3-butanediol by almost 2.7-fold compared to that of the wild type and EC∆ldhA strains (Table 1). However, small amounts of 2,3-butanediol were still detected in a few of the EC∆budC and EC∆budC∆ldhA strains, indicating the presence of other genes encoding enzymes that convert acetoin to 2,3-butanediol in *E. cloacae*.

Theoretically, the formation of 1 mol succinic acid from glucose requires 1 mol of CO_2_ [20, 28]. Therefore, CO_2_ is indispensable for succinic acid biosynthesis, and many studies have demonstrated that succinic acid production can be increased by adding HCO_3_^−^ to the fermentation medium [12, 28]. Cheng et al. [28] increased succinic acid production in *K. pneumoniae* by adding HCO_3_^−^ to the fermentation medium. In another study, Wu et al. [20] reported yields of 40.67 g L^−1^ 2,3-butanediol and 21.79 g L^−1^ succinic acid by adding NaHCO_3_ to *E. cloacae*. In this study, supplying NaHCO_3_ during batch fermentation may enhance succinic acid production by improving the quantity of dissolved CO_2_ and by increasing the carbon flux to succinic acid. When grown in fermentation medium without NaHCO_3_, the final acetoin production (16.45 g L^−1^) was slightly higher; however, the final amount of succinic acid produced was only 1.15 g L^−1^. When grown in fermentation medium supplemented with NaHCO_3_, succinic acid production was 34.8% higher than the amount produced during batch fermentation without NaHCO_3_ (Table 2).

In general, the production of succinic acid was higher under anaerobic conditions, and bacterial producers of succinic acid can be found among facultative and strictly anaerobic rumen bacteria such as *Mannheimia succiniciproducens* [29], *Actinobacillus succinogenes* [30], and *Anaerobiospirillum succiniciproducens* [31]. *E. cloacae* is a facultative anaerobe, and when it is cultured under anaerobic conditions, the glucose consumption rate of the ΔbudCΔldhA strain is slower, resulting in lower production concentration of acetoin. In addition, when cultured under anaerobic conditions, the ΔbudCΔldhA strain was found to produce lactic acid (Additional file 1: Fig. S2). Although we only knocked out d-lactate dehydrogenase, this may activate other lactate dehydrogenases under anaerobic conditions, such as l-lactate dehydrogenase, leading to the production of lactic acid.

A previous study showed that reducing the carbon flux to lactate, ethanol, and acetate by-products can be performed by deleting the *ldhA*, *adhE*, and *pta* genes in *K. pneumoniae* [32]. In this study, by blocking lactic acid synthesis pathways to redirect more carbon sources to succinic acid synthesis in wild type *E. cloacae*, the engineered EC∆budC significantly increased succinic acid yield. This engineering approach may represent a practical strategy involving the deletion of *ldhA* and *budC* genes to reduce carbon flux towards the formation of by-products.

## Conclusions

In this study, we engineered an *E. cloacae budC* and *ldhA* double-deletion strain (namely, EC∆budC∆ldhA) to produce succinic acid and acetoin. The highest acetoin and succinic acid titers achieved by this engineered strain were 39.5 and 20.3 g L^−1^, respectively, during optimization of fed-batch fermentation conditions. Our findings demonstrated that the EC∆budC∆ldhA strain would be useful for the simultaneous production of commercial products (acetoin and succinic acid) and the prevention of by-product formation, thus reducing the cost of microbial fermentation in a single step.

## Methods

### Bacterial strains

The strains used in this study are described in Table 3. *Escherichia coli* and *E. cloacae* were grown in LB broth with rotary shaking agitation at 200 rpm at 37 °C and 35 °C, respectively. Ampicillin (100 µg mL^−1^) and kanamycin (50 µg mL^−1^) were added to LB broth. *E. cloacae* (CICC 10011) was purchased from the China Center of Industrial Culture Collection (China). *E. coli* DH5α was used as the host for all recombinant plasmid constructs. *E. coli* S17-1 λpir, which is able to host pKR6K and its derivatives, was used for conjugation with *E. cloacae*.

**Table 3:** Strains and plasmid used in this study

Name	Relevant genotype	Source and ref
Strains		
DH5α	F^−^φ80 lac ZΔM15 Δ(lacZYA-arg F) U169 endA1 recA1 hsdR17(rk^−^,mk^+^) supE44λ^−^ thi -1 gyrA96 relA1 phoA, Recipient in transformations	Sangon Biotech
S17-1 λpir	RP4-2(Km::Tn7,Tc::Mu-1) pro-82 LAMpir recA1 endA1 thiE1 hsdR17 creC510; conjugative strain able to host *-pir*-dependent plasmids	Zoman Biotechnology
* E. cloacae* CICC 10011	Wild type	CICC
EC∆budC	*E. cloacae* CICC 10011 budC disruption mutant strain	This study
EC∆ldhA	*E. cloacae* CICC 10011 ldhA disruption mutant strain	This study
EC∆budC∆ldhA	*E. cloacae* CICC 10011 budC and ldhA disruption mutant strain	This study
Plasmid		
pGEM-T Easy Vector	Cloning vector, Ap^r^	Promega
pToriR6K	pGEM-T Easy Vector with 0.6-kb oriR6K fragment, Ap^r^	This study
pTBCup	pGEM-T Easy Vector with 0.5-kb *budC* upstream fragment, Ap^r^	This study
pTBCdown	pGEM-T Easy Vector with 0.5-kb *budC* downstream fragment, Ap^r^	This study
pTLAup	pGEM-T Easy Vector with 0.5-kb *ldhA* upstream fragment, Ap^r^	This study
pTLAdown	pGEM-T Easy Vector with 0.5-kb *ldhA* downstream fragment, Ap^r^	This study
pK18*mobSacB*	*oriT*(RP4) *sacB lacZα* Plac Pmbi; mobilization and counterselection, Kan^r^	BCRC
pRL27	Mini-Tn5 transposon (oriR6K) delivery vector, Kan^r^	[33]
pKR6K	R6K replicon; gene replacement vector, Kan^r^	This study
pKΔbudC	pKR6K derivative, carries a 771 bp deletion of *budC*, Kan^r^	This study
pK∆ldhA	pKR6K derivative, carries a 990 bp deletion of *ldhA*, Kan^r^	This study

CICC, The China Center of Industrial Culture Collection, China; BCRC, The Bioresource Collection and Research Centre, FIRDI, Taiwan

### Plasmid construction

Plasmids constructed and used are described in Table 3. The gene replacement vector of *E. cloacae* was constructed by a previously described method [22]. The R6K origin of replication was amplified with primers (BspHI-oriR6K-F and BsaXI-oriR6K-R) using the plasmid pRL27 as a template. The 0.6 kb oriR6K fragment was ligated to the pGEM-T Easy vector (Promega, Madison, WI, USA) to create the plasmid pToriR6K. The oriR6K fragment (BspHI/BsaXI) was digested from pToriR6K and cloned into plasmid pK18mobsacB to create the suicide plasmid pKR6K. The plasmid pKR6K was used for gene knockout by homologous recombination in *E. cloacae*.

Gene knockout mutants of *E. cloacae* were constructed using the suicide vector pKR6K. To construct the *budC* and *ldhA* gene replacement vector of *E. cloacae*, the selected flanks were 510 bp long and homologous to sequences upstream and downstream of the region targeted for deletion. The upstream and downstream flanking sequences of *the budC* and *ldhA* genes were amplified with their respective primers (EcoRI-budCup-F/EcoRI-budCup-R, XbaI-budCdown-F/SphI-budCdown-R and EcoRI-ldhAup-F/BamHI-EcoRI-ldhAup-R, XbaI-ldhAdown-F/SphI-SalI-ldhAdown-R) using the total genomic DNA of *E. cloacae* as a template for PCR and cloned into the pGEM-T Easy vector to generate plasmids pTBCup and pTBCdown. Then, the budC upstream and downstream fragments were digested by *Eco*RI and *Xba*I/*Sph*I from plasmids pTBCup and pTBCdown, respectively. The two fragments were ligated to pKR6K digested with *Eco*RI and *Xba*I/*Sph*I, producing pKΔbudC. The ldhA upstream and downstream fragments were digested by *Eco*RI and *Xba*I/*Sal*I from plasmids pTLAup and pTLAdown, respectively. The two fragments were ligated to pKR6K digested with *Eco*RI and *Xba*I/*Sal*I, producing pKΔldhA. Then, the plasmids pKΔbudC and pKΔldhA were transformed into *E. coli* S17-1. *E. coli* S17-1 (pKΔbudC and pKΔldhA) was used as the donor in conjugation with *E. cloacae*. The primer sequences are shown in Table 4.

**Table 4:** Primers used in this study

Primer	Sequence	References or source
BspHI-oriR6K-F	5′-aatttcatgacagttcaacctgttgatagtac-3′	[23]
BsaXI-oriR6K-R	5′-aattggagaggcggtagagagagacaatgtcagccgttaagtgttc-3′	[23]
EcoRI-budCup-F	5′-aattgaattcagcttccacatctggatcgcccgct-3′	This study
EcoRI-budCup-R	5′-aattgaattcttctctgtccttatagtgagtcaca-3′	This study
XbaI-budCdown-F	5′-aatttctagataaattctaataagctctgacatga-3′	This study
SphI-budCdown-R	5′-aattgcatgccttcatcgtgcgcatttcgcccggc-3′	This study
budC-up-F	5′-aggacatcgtcaataacgacgtgac-3′	This study
budC-down-R	5′-ttcatcttcggtaaagatcagcgtg-3′	This study
budC-F	5′-atgcaaaaagttgctctcgtaaccg-3′	This study
budC-R	5′-ttagttgaacaccatcccaccatca-3′	This study
EcoRI-ldhAup-F	5′ –aattgaattcaccgtgttaagttcaagcgcaccaa-3′	This study
BamHI-EcoRI-ldhAup-R	5′-aattggatccgaattcaagactttctccagtgattttacat-3′	This study
XbaI- ldhAdown-F	5′-aatttctagagccgacatgccgggtggcggttacg-3′	This study
SphI-SalI-ldhAdown-R	5′-aattgcatgcgtcgacggcgacggtcattatttcgcaggcg-3′	This study
ldhA-up-F	5′-tttttggcgcaacggttgacggtgc-3′	This study
ldhA-down-R	5′-atgcgggtcgccgccgcgcctgcca-3′	This study
ldhA-F	5′-atgaaactcgcggtatatagcacaa-3′	This study
ldhA-R	5′-ttagactatctcgttaggacacgct-3′	This study

### Gene knockout in the chromosome of *E. cloacae*

Allelic exchange of *E. cloacae* was performed as previously described [10] with slight modifications. The constructed strains used are described in Table 3. Strain EC∆budC was constructed by allelic exchange of plasmid pKΔbudC into *E. cloacae*. Strain EC∆ldhA was constructed by allelic exchange of plasmid pKΔldhA into *E. cloacae*. Strain EC∆budC∆ldhA was constructed by allelic exchange of plasmid pKΔldhA into *E. cloacae* strain EC∆budC. Colonies with confirmed deletions were screened by PCR using specific primers. The primer sequences are shown in Table 4.

### Batch and fed-batch fermentations

The seed culture, batch fermentation, and fed-batch fermentation were carried out according to the procedure described by Wu et al. [20]. Sterilized glucose was added before fermentation. Wild type and gene knockout strains of *E. cloacae* were inoculated into flasks (250 mL) containing 50 mL of seed culture medium and cultured overnight at 35 ℃ with continuous shaking at 150 rpm. The fermentation medium contained final concentrations of 5% (v/v) seed medium.

Batch fermentation and fed-batch fermentation were conducted in 250 mL flasks containing 50 mL of medium. Cultivation was carried out at 35 °C with a speed at 150 rpm. The pH was maintained by the addition of NaHCO_3_. Samples were withdrawn periodically to measure the OD_600_ and the concentrations of glucose, succinic acid, acetoin, and by-products. Each experiment described in this research was performed in two replicates.

### Fed-batch fermentations in the bioreactor

Seed culture (5%, v/v) was inoculated into the fermentation medium, and fed-batch fermentation was carried out in a 3-L stirred-vessel bioreactor (BLBIO-3GC, Bailun, China) containing 1.5-L of fermentation medium under 0.5 vvm air flow. Cultivation was performed at 35 °C with a speed at 300 rpm and an aeration rate of 0.5 vvm. The pH was maintained by the addition of NaHCO_3_.

### Analytical methods

Glucose, succinic acid, acetoin, and by-products were analyzed by the methods described in Wu et al. [20]. Samples were measured by HPLC (LC20, Shimadzu, Japan) using an Aminex HPX-87H column (Bio Rad, USA) with a refractive index detector (RID-20A).

## Supplementary Information

**Additional file 1: Fig. S1.** Time course of fed-batch fermentation of EC∆budC under the optimized conditions. **Fig. S2.** Time course of batch anaerobic fermentation of EC∆budC∆ldhA.

## Data Availability

The datasets supporting the conclusions of this article are included within the article.
