# Supplementary material for: Co-production of acetoin and succinic acid by metabolically engineered Enterobacter cloacae
Source: Biotechnol Biofuels. 2021 Jan 19;14:26. doi: 10.1186/s13068-021-01878-1 (PMC7816431; doi:10.1186/s13068-021-01878-1)
Supplement: Supplementary file 1 — Additional file 1: Fig. S1. Time course of fed-batch fermentation of EC∆budC under the optimized conditions. Fig. S2. Time course of batch anaerobic fermentation of EC∆budC∆ldhA. [file 13068_2021_1878_MOESM1_ESM.docx]

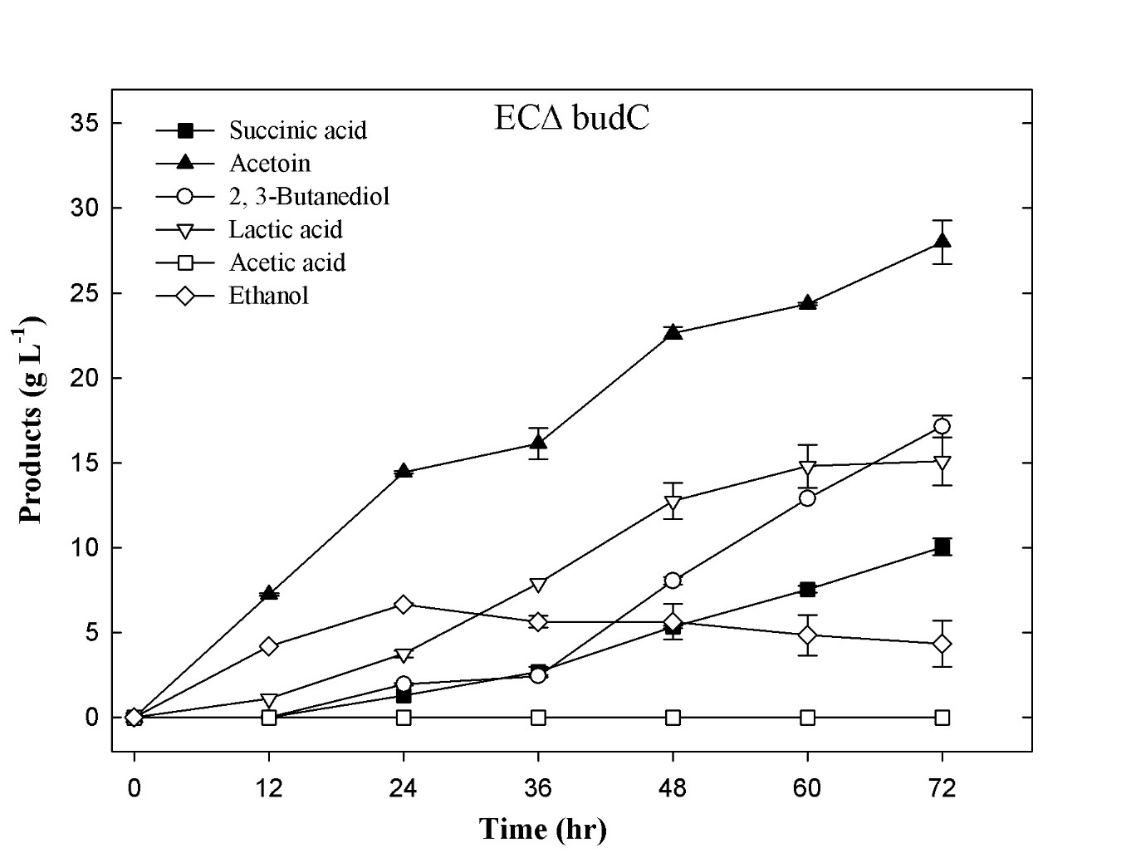


**Fig. S1** Time course of fed-batch fermentation of EC∆budC under the optimized conditions. (A) Glucose, acetoin, succinic acid, (B) by-product. The experiments were conducted in 50 mL of fermentation medium containing 60 g L^-1^ glucose in a 250 mL flask. NaHCO_3_ (2.5, 1, 1, 5, 5, 5 and 2 g L^-1^) was added at 0, 6, 12, 24, 36, 48, and 60 hr of fermentation, respectively. Glucose (60, 25, 35, 35, and 35 g L^-1^) was added at 0, 12, 24, 36, and 48 hr of fermentation, respectively. Samples were withdrawn every 12 hr for detection of cell density and concentration of substrates and products.

***
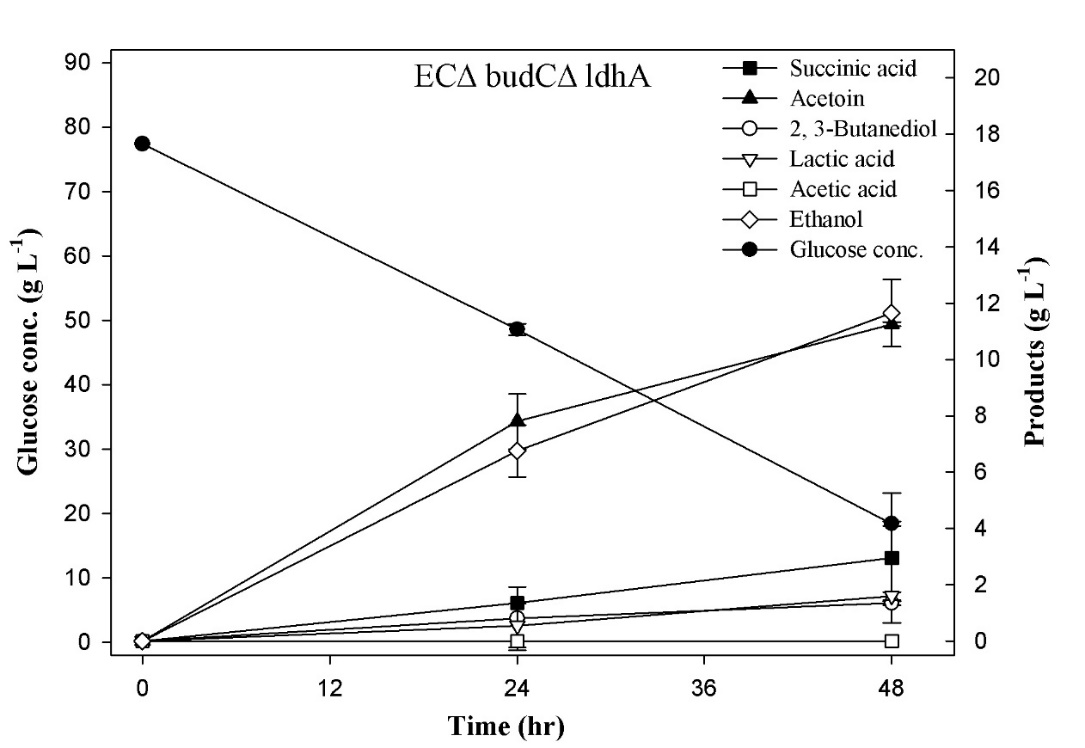
***

**Fig. S2** Time course of batch anaerobic fermentation of EC∆budC∆ldhA.

The experiments were conducted in 50 mL of fermentation medium containing 80 g L^-1^ glucose and 5.0 g L^-1^ NaHCO_3_ in a 250 mL flask at 35 °C with shaking (150 rpm).
